# Resistance exercise breaks during prolonged sitting augment the blood flow response to a subsequent oral glucose load in sedentary adults

**DOI:** 10.1113/EP091535

**Published:** 2024-08-02

**Authors:** Emily M. Rogers, Nile F. Banks, Emma R. Trachta, Morgan S. Wolf, Alexander C. Berry, Anna E. Stanhewicz, Lucas J. Carr, Bethany Barone Gibbs, Nathaniel D. M. Jenkins

**Affiliations:** ^1^ Department of Health and Human Physiology The University of Iowa Iowa City Iowa USA; ^2^ Department of Kinesiology The University of Wisconsin Madison Wisconsin USA; ^3^ Department of Epidemiology and Biostatistics West Virginia University School of Public Health Morgantown West Virginia USA; ^4^ Abboud Cardiovascular Research Center The University of Iowa Iowa City Iowa USA; ^5^ Fraternal Order of Eagles Diabetes Research Center The University of Iowa Iowa City USA

**Keywords:** exercise breaks, sedentary behaviour, vascular insulin sensitivity

## Abstract

Sitting‐induced impairments in postprandial blood flow are an important link between sedentary behaviour and cardiometabolic disease risk. The objective of this work was to examine the effects of resistance exercise breaks (REB) performed every 30 min during an otherwise sedentary 3‐h period on the vasodilatory response to a subsequent oral glucose load in sedentary adults. Twenty‐four sedentary adults (27 ± 7 years, 16 females) completed two conditions. Fasting blood glucose, insulin, popliteal artery blood flow (PABF) and gastrocnemius perfusion were measured immediately before standardized breakfast consumption. After breakfast, the 3‐h REB or uninterrupted (SIT) intervention period commenced. Participants sat at a workstation, and popliteal artery shear rate (PASR) was measured 60 and 120 min into this period. In the REB condition, participants performed a 3‐min REB (3 × [20 s squats, 20 s high knees, 20 s calf raises]) every 30 min. Following the intervention period, baseline measurements were repeated. Participants then consumed a 75 g glucose beverage, and PABF and perfusion were measured every 30–60 min for the following 120 min. Relative to SIT, REB increased PASR at 60 min (+31.4 ± 9.2/s, *P *= 0.037) and 120 min (+37.4 ± 10.2/s, *P *= 0.019) into the intervention period. Insulin and glucose increased (*P <* 0.001) in response to glucose consumption, with no differences between conditions (*P *≥ 0.299). In response to the glucose load, perfusion (1.57 vs. 1.11 mL/100 mL/min, *P *= 0.023) and PABF (+45.3 ± 11.8 mL/min, *P *= 0.001) were greater after REB versus SIT. Performing 3‐min REB every 30 min during an otherwise sedentary 3‐h period augmented leg blood flow responses to an oral glucose load.

## Abstract (highlights)

- **What is the central question of this study?**
  Can 3‐min resistance exercise breaks (REB) performed during an otherwise sedentary 3‐h period augment the vasodilatory response to a subsequent oral glucose load in sedentary adults?
- **What is the main finding and its importance?**
  Performing 3‐min REB, which included squats, high knees, and calf raises, every 30 min augmented lower limb blood flow responses to a subsequent oral glucose load compared to 3 h of uninterrupted sitting in sedentary adults. Sitting‐induced impairment in postprandial vasodilatory function has been identified as a link between sedentary behaviour and cardiometabolic disease. Thus, the current study presents a potentially effective strategy to offset this risk.

## 1 INTRODUCTION

Sedentary behaviour time has increased significantly over the last few decades as a result of increased sitting time in the workplace, during transportation, and in social settings (Church et al., 2011). Currently, >80% of jobs in the USA are primarily sedentary (Gremaud et al., 2018). Employees working in these sedentary jobs spend >75% of their workday sitting (Gremaud et al., 2018), and sedentary behaviour is now recognized as an independent risk factor for cardiovascular and metabolic disease (CVD) and all‐cause mortality (Joseph et al., 2016; Katzmarzyk et al., 2020; Pandey et al., 2016). Indeed, prolonged sitting time directly correlates with subclinical coronary atherosclerosis severity (Lim et al., 2022) and insulin resistance (Kim et al., 2018; Parker et al., 2023).

Stimulated increases in skeletal muscle blood flow are critical for nutrient disposal and hormone delivery in the postprandial state (Tamariz‐Ellemann et al., 2022). Impairments in the blood flow response to meal ingestion are thought to serve as a link between CVD and metabolic diseases (Padilla et al., 2015). Insulin is the primary hormone that serves to couple the regulation of metabolic and haemodynamic homeostasis via its vasodilatory effects to direct blood flow to nutritive capillary beds in response to nutrient ingestion (Banks et al., 2022; Clark et al., 2003; Scherrer et al., 1994; Steinberg et al., 1994; Timmerman et al., 2010) and there is a functional link between insulin‐mediated skeletal muscle vasodilatation and insulin‐mediated glucose uptake (Banks et al., 2022; Cleland et al., 1999). Notably, impairments in insulin‐stimulated blood flow have been demonstrated after just 2 h of uninterrupted sitting in healthy adults (Walsh et al., 2019), an effect that appears to be caused by an increase in the balance of Ras–mitogen‐activated protein kinase–endothelin‐1 (ET‐1) versus the phosphatidylinositol 3‐kinase–nitric oxide pathway in response to insulin signalling due to prolonged exposure to reduced laminar shear stress patterns (Olver et al., 2019b; Padilla et al., 2015; Walsh et al., 2019). However, acute exercise augments insulin‐mediated increases in skeletal muscle blood flow via a nitric oxide‐dependent mechanism (Sjoberg et al., 2017), and short‐term exercise training augments skeletal muscle blood flow in response to an oral glucose load in association with improvements in glycaemic control in sedentary individuals with type 2 diabetes (T2D) (Mikus et al., 2011; Russell et al., 2017). Thus, these findings support an important link between postprandial skeletal muscle blood flow and glycaemic control (Baron et al., 1996) that is disrupted in response to sedentary behaviour. However, manipulating endothelial shear stress patterns during otherwise sedentary periods may be an effective strategy to augment nutrient‐stimulated skeletal muscle blood flow and ultimately reduce sedentary behaviour‐related risk for CVD and T2D.

Shear stress decreases during prolonged sitting (Morishima et al., 2016; Walsh et al., 2019) but can be increased by stimulating blood flow, such as during exercise (Morishima et al., 2016; Tinken et al., 2010). Resistance exercise breaks (REB) transiently increase the shear rate and counteract impairments in flow‐mediated dilatation (FMD) in the face of prolonged sitting (Climie et al., 2018). However, whether REB can counter decrements in vasodilatory responsiveness to a glucose load caused by prolonged sedentary periods is undetermined. It is also unclear whether the effects of sitting on blood flow responses to glucose ingestion are sex‐specific in young adults. Recent evidence suggests that the female sex (pre‐menopause) affords protection against impairments in insulin‐stimulated blood flow in response to the adoption of a short‐term obesogenic lifestyle, which involved reducing physical activity and over‐consuming sugar‐sweetened beverages for 10 days (Smith et al., 2022). However, no studies to date have explored whether the protective effect of female sex extends to the effects of prolonged sitting on the blood flow response to a subsequent oral glucose load.

In the current study, we examined the effects of REB performed every 30 min during an otherwise sedentary 3‐h period on the vasodilatory response to a subsequent oral glucose challenge in sedentary adults. We also explored whether the effects of REB versus SIT on blood flow responses to an oral glucose load were dependent on biological sex. We hypothesized that (1) REB would augment blood flow responses to oral glucose ingestion following an otherwise sedentary period in young adults and (2) the 3‐h SIT condition would elicit a greater decrease in the blood flow response to oral glucose ingestion in males compared to females; thus, the blood flow response would be augmented more in response to REB in males versus females.

## 2 METHODS

### 2.1 Ethical approval

Informed consent was obtained electronically using the Research Electronic Data Capture (REDCap) system. This study conformed to the standards set by the *Declaration of Helsinki*, except for registration in a database, and was approved by, and carried out in accordance with, the University of Iowa's Institutional Review Board for the protection of human subjects (IRB Approval no. 202212166, approval date: 14 January 2023).

### 2.2 Participants

Participant characteristics are displayed in Table 1. Twenty‐four sedentary young adults (age range 18−42 years) completed this study (16 females, 8 males). Participants were recruited via campus‐wide email and word of mouth. Initial eligibility screening was performed, and health history questionnaires were completed on REDCap. An International hysical Activity (PA) Questionnaire Short Form (IPAQ‐SF) was also completed to assess participants' habitual PA levels. Inclusion/exclusion criteria were established a priori and were chosen to balance internal validity while maximizing external validity to the population of interest (sedentary adults). Therefore, to be eligible, participants must have met the following criteria: were aged 18−45 years; were free from cardiometabolic disease (CMD); had a fasting blood glucose <126 mg/dL (measured via finger stick), indicating that they were free from diabetes (American Diabetes Association, 2020); had a body mass index (BMI) <30 kg/m^2^, indicating that they did not have class 1 obesity; were sedentary (defined as sitting for at least 7 h/day on weekdays); were ready to begin a PA programme without additional need for medical clearance as determined by the PA readiness questionnaire+ (PAR‐Q+); and were able to complete the REB exercises without pain. Participants were excluded if they were engaging in >60 min of moderate aerobic exercise each day, as this level of PA has been shown to be protective against sedentary behaviour‐induced risk (Ekelund et al., 2016); if they were pregnant or peri/postmenopausal; or if they were taking any hypoglycaemic medications. All potentially eligible participants were contacted by a researcher and were scheduled to come to the lab for an in‐person screening visit. At this visit, participants had their fasting blood glucose and BMI measured to confirm that values were under 126 mg/dL and 30 kg/m^2^, respectively. Participants also performed a round of REB to screen for any pain and to become familiarized with the movements before their first experimental visit. If participants remained eligible after screening, they were scheduled for their two 6‐h experimental visits. According to fasting blood glucose values, five participants had prediabetes (i.e., fasting blood glucose values of 100−125 mg/dL). We conducted a sensitivity analysis to confirm that prediabetes status did not influence our findings.

**TABLE 1:** Participant characteristics.

	All participants (*n* = 24)	Females (*n* = 16)	Males (*n* = 8)	*P*
Age (years)	27 ± 7	29 ± 7	22 ± 4	**0.007**
Race (*n*, %)				
White	16, 67%	13, 81%	3, 37%	
Asian	7, 29%	3, 19%	4, 50%	
More than one race	1, 4%	0, 0%	1, 13%	
Height (cm)	169 ± 10	165 ± 9	179 ± 6	**<0.001**
Weight (kg)	70 ± 11	68 ± 10	75 ± 12	0.199
BMI (kg/m^2^)	24 ± 3	25 ± 3	23 ± 3	0.235
SBP (mmHg)	109 ± 9	108 ± 8	111 ± 11	0.495
DBP (mmHg)	69 ± 9	71 ± 8	66 ± 10	0.322
Physical activity (MET min/week)	329 ± 204	335 ± 233	318 ± 167	0.866
Habitual calorie consumption (Kcal/day)	1636 ± 589	1485 ± 241	1981 ± 792	0.159
Fasting serum insulin (μIU/mL)	17 ± 11	15 ± 7	20 ± 14	0.287
Fasting blood glucose (mg/dL)	94 ± 8	93 ± 9	94 ± 8	0.835

*Note*: Data are presented here as means ± SD except for race which is presented as *n* and % of total group. Where appropriate, the average of participants’ fasting values from the two experimental visits were calculated and are presented here. *P*‐values are from Student's unpaired *t*‐test examining differences between female and male values. Significant *P*‐values (<0.05) are shown in bold. Abbreviations: BMI, body mass index; DBP, diastolic blood pressure; SBP, systolic blood pressure.

### 2.3 Experimental design

**FIGURE 1:**
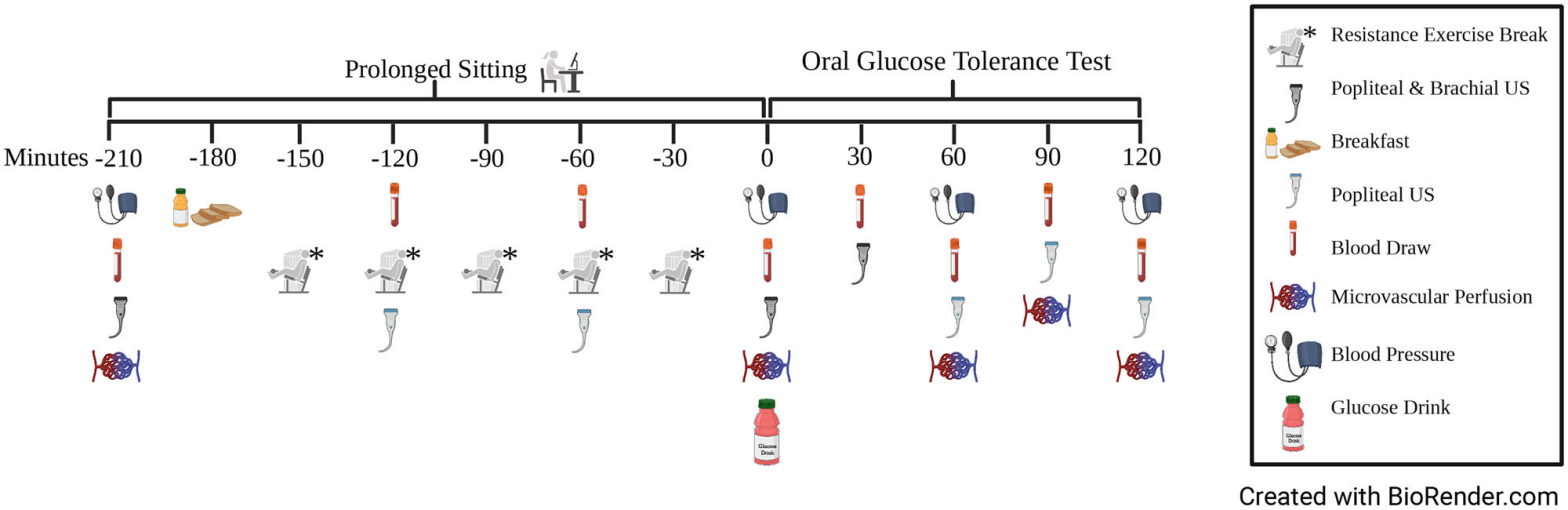
Timeline of events during experimental visits. From min −180 to 0, participants underwent the SIT or the REB intervention. During REB, they performed 3‐min exercise breaks (3 × [20 s squats, 20 s high knees with gluteal contractions, and 20 s calf raises]) every 30 min, while during SIT they remained sedentary in the seated position for the entire 3‐h period. *Was only performed at the REB condition. US, ultrasound.

This study employed a randomized cross‐over design, where each participant completed a 3‐h sedentary condition (SIT) and a 3‐h sedentary condition with REB performed every 30 min (REB). After these 3‐h periods, blood flow responses to oral glucose ingestion were assessed. The visit order was randomized using the flip of a coin once the subject was enrolled in the study; heads meant REB would be first and tails meant SIT would be first. An illustration of the study design and experimental visits is displayed in Figure 1. Experimental visits were separated by 3−7 days. All visits started between 05.00 and 12.00 h, and start‐times were consistent (±2 h) within participants. Participants were asked to fast from all food and drink except water for 10 h, to avoid caffeine consumption for 12 h, and to abstain from exercise for 24 h before each visit. Upon arrival to the lab, an intravenous (i.v.) catheter was placed in the participants’ antecubital vein, and baseline blood samples were taken to assess glucose, insulin, and ET‐1 levels. This i.v. catheter remained in the participants’ vessel until the end of the visit. Participants then rested supine on a bed for 10 min before their blood pressure was measured. After this, gastrocnemius perfusion and brachial and popliteal artery blood flow were measured. The arm and leg that were used for blood flow analyses remained consistent between visits within participants. Participants then consumed a standardized breakfast in a 10 min period. Breakfast consisted of a breakfast bar (Kind Breakfast Protein Bar, Chocolate) with orange juice (296 mL; Tropicana 100% Orange Juice) providing a total of 350 kcal (20% fat, 70% carbohydrates, 10% protein). At this time, the participant and ultrasonographer were told which condition the participant had been randomized to, and the 3‐h intervention period began. During the SIT condition, participants remained seated for 3 h. During the REB condition, participants stood up every 30 min to complete a 3‐min bout of REB. After completing the 3‐h intervention, participants rested supine for 10 min before a blood sample was taken and blood pressure, perfusion, and blood flow were measured. Participants then consumed a 75‐g glucose beverage to examine the effects of REB versus SIT on the blood flow response to glucose ingestion. Following glucose consumption, participants lay supine for the subsequent 120 min. During this period, vascular (i.e., perfusion and blood flow) measures and blood samples were obtained every 30−60 min.

This experimental design allowed us to observe changes in our dependent variables in response to 3 h of sitting with and without REB both (a) before glucose consumption (i.e., from min −210−0) and (b) after glucose consumption (i.e., from min 0−120). Throughout this paper, we will refer to the effects of the REB versus SIT condition (a) before glucose consumption as ‘in response to’ or ‘during’ ‘the intervention period’ and (b) after glucose consumption as ‘in response to an oral glucose load’ or similar.

### 2.4 Resistance exercise break and sedentary behaviour intervention

During the SIT condition, participants sat continuously without interruption and were allowed to work, read or watch TV for 3 h. Participants were asked to perform the same type of sedentary work/activity at both visits to maintain consistency. During the REB condition, participants completed a REB every 30 min throughout the otherwise sedentary period. The REB consisted of three sets of 20 s of bodyweight squats, 20 s of high knees with gluteal contractions, and 20 s of calf raises at a tempo of one repetition every 2 s, as previously described (Climie et al., 2018; Dempsey et al., 2016a, 2016b). This lower‐body‐only exercise break protocol was chosen as it allowed us to assess the local (i.e., popliteal) and systemic (i.e., brachial) vasodilatory effects of REB in response to the oral glucose challenge. To avoid any non‐protocol‐related muscle contraction during experimental visits, participants were not allowed to leave their seats for any reason outside of the study protocol. When needed, participants were brought to the restroom in a wheelchair by a researcher.

### 2.5 Lifestyle controls

Participants were asked to eat foods consistent with their habitual diet on the days before each visit and to eat the same foods on the days preceding both visits. Food consumption for these days was recorded on food logs. This information was then entered into ESHA's Food Processor® nutrition analysis Software (https://www.esha.com, ESHA Research, Oak Brook, IL, USA), which provided the number of kcal consumed by each participant on each pre‐experimental day.

After consenting and prior to scheduling, female participants were asked several questions on the health history questionnaire to obtain information regarding contraceptive use, type, and timing, as well as menstrual cycle status, duration, and timing. Participant visits were scheduled so that each individual had both of their experimental visits within the same estimated phase of their cycle, as the effect of the menstrual cycle phase on vascular function remains unclear (Stanhewicz & Wong, 2020; Wenner & Stachenfeld, 2020). This meant that the majority of female participants’ visits were separated by 3−4 days in an attempt to complete both experimental visits such that there was minimal variation in circulating sex hormones between visits. During both experimental visits, females were asked if they were menstruating that day and when their most recent menstruation began in order to confirm that their predicted menstrual cycle phase remained the same.

### 2.6 Gastrocnemius perfusion

Perfusion was measured using near‐infrared spectroscopy with the venous occlusion technique (NIRS‐VOT). This technique is reliable, displaying an intraday coefficient of variation of 10% ± 5.5% (De Blasi et al., 1994), and is more accessible than but highly correlated (*r* = 0.93) with the gold standard venous occlusion strain‐gauge plethysmography (VOP) method (De Blasi et al., 1994). Further, intrasubject correlation coefficients comparing NIRS‐VOT and VOP‐derived perfusion are 0.85−0.98, and the associated standard error of estimates is 0.234−0.986 (Homma et al., 1996). Finally, NIRS‐VOT‐ and VOP‐derived measurements of perfusion respond similarly to physiological perturbations (Homma et al., 1996).

B‐mode ultrasound was used to detect the thickest part of the gastrocnemius medialis on each participant's leg. The NIRS device (PortaMon, Artinis Medical Systems, Einsteinweg, The Netherlands) was placed on this part of the muscle and was covered with an optically dense cloth to eliminate light interference (Rogers et al., 2023). Participants were fitted with a segmental cuff on their upper thigh and were left to rest supine for 10 min before NIRS data acquisition began. Total haemoglobin (tHb) values were visualized on a laptop using OxySoft software (Artinis Medical Systems). To correct for photon scattering, the differential path‐length factor length was set to 5.8 in OxySoft. After 10 min of rest, 1 min of steady baseline tHb data was acquired before the thigh cuff was inflated. The thigh cuff was inflated rapidly to a pressure of 70–80 mmHg to ensure interruption of venous flow without affecting arterial inflow. Muscle perfusion of the gastrocnemius medialis (in mL/100 mL/min) was determined via the linear increase in tHb following cuff inflation, using the following equation (De Blasi et al., 1994; Girardis et al., 2003):

$$\mathrm{Perfusion}\;\left(\mathrm{mL}/100\;\mathrm{mL}/\min\right)=1/C\times \left[\Delta t\mathrm{Hb}/\Delta t\right]\times 60$$

where, *t* is time (s) and *C* is blood haemoglobin concentration, which was assumed to be 7.5 mmol/L for females and 8.5 mmol/L for males (Lucero et al., 2018).

### 2.7 Brachial and popliteal artery blood flow and shear rate

Following the NIRS‐VOT assessment, the brachial and then popliteal arteries were visualized using high‐resolution duplex ultrasonography (NextGen LOGIQ e, GE Medical Systems, Milwaukee, WI, USA) and a 12 MHz linear array transducer (GE L4‐12t‐RS, GE Medical Systems). The insonation angle was set at 60° to the artery wall, the correction angle was positioned parallel to the vessel walls, and the pulse wave gain was set at 33 Hz. The sample volume gates were set as wide as possible, without including the artery walls, to obtain a sample volume across the entire diameter of the artery. Once a clear image was acquired, a 1‐min video clip of the artery on the ultrasound screen was obtained using a high‐resolution screen capture device (AV.io HD, Epiphan Systems, Inc., Palo Alto, CA, USA). This video was later analysed offline using specialized edge detection software (Cardiovascular Suite, Quipu srl., Pisa, Italy), which measured continuous artery diameter, blood flow velocity, and shear rate. Popliteal shear rate was measured at baseline and every 60 min during the 3‐h intervention period to determine the effects of REB on shear rate. To calculate blood flow, the artery cross‐sectional area was first calculated as [π × (artery diameter/2)^2^], and then the following equation was used:

$$\begin{array}{ccc} \mathrm{Blood}\;\mathrm{Flow}\left(\mathrm{mL}/\min\right) & = & \left(\mathrm{blood}\;\mathrm{flow}\;\mathrm{velocity}\;\left(\mathrm{cm}/s\right)\right.\\ & & \left.\times \;\mathrm{artery}\;\mathrm{cross}\;\mathrm{sectional}\;\mathrm{area}\left(cm^{2}\right)\right)\times 60 \end{array}$$

The shear rate was calculated using the following equation:

$$\begin{array}{ccc} \mathrm{Shear}\;\mathrm{Rate} & = & \left(4\times \mathrm{blood}\;\mathrm{flow}\;\mathrm{velocity}\left(\mathrm{cm}/s\right)/\mathrm{artery}\;\mathrm{diameter}\left(\mathrm{cm}\right)\right)\\ & & \left(\mathrm{Thijssen}\;\mathrm{et}\mathrm{al}.,2019\right) \end{array}$$

### 2.8 Glucose beverage consumption and blood sampling and analysis

After completing the 3‐h intervention period, participants consumed a beverage containing 75 g of glucose (Fisherbrand Glucose Tolerance Test Beverage, Thermo Fisher Scientific, Waltham, MA, USA) to induce a dramatic increase in endogenous insulin release. Venous blood samples were taken at fasting (−210 min) as well as at the −120‐, −60‐, 0‐, 30‐, 60‐, 90‐, and 120‐min time points to assess glucose, insulin, and ET‐1. At each of these time points, whole blood glucose was measured immediately in duplicate (or in triplicate if the difference between the first two readings was ≥7 mg/dL), using a StatStrip Xpress 2 Glucose Meter (Nova Biomedical, Waltham, MA, USA). The average of these readings was recorded and used for analyses. Serum was also obtained using serum separator tubes (BD Vacutainer, Becton Dickinson and Company, Franklin Lakes, NJ, USA). The tubes were inverted immediately after collecting whole blood and were left for 30 min to clot. After centrifugation at 1000 *g* for 10 min, separated serum was aliquoted into 1.5 mL microcentrifuge tubes and stored in a −80°C freezer (SU780XLE, Stirling Ultracold, Athens, OH, USA) for later analyses of insulin and ET‐1. Due to difficulties with catheter placement and/or catheter failure, data derived from blood samples were available for 18 of our 24 participants (*n* = 10 females and *n* = 8 males).

A sandwich enzyme‐linked immunosorbent assay (ELISA) kit (Human Insulin ELISA, cat. no. KAP1251, BioVender, Karasek, Czech Republic) was used to measure insulin. This kit had a 5.1−250 μIU/mL detection range and a sensitivity of 0.17 μIU/mL. The inter‐assay coefficient of variation (CV) was ≤10%. The plate was read using a microplate photometer (Multiskan FC Microplate Photometer, Thermo Fisher Scientific). ET‐1 concentrations were measured using an ELISA kit (Human Endothelin‐1, QuantiGlo ELISA, cat. no. QET00B, R&D Systems Inc., Minneapolis, MN, USA) with a detection range of 0.343−250 pg/mL and a sensitivity of 0.064 pg/mL. The inter‐assay CV was ≤18%. This plate was read using a Synergy LX Multi‐Mode Microplate Reader (Agilent Technologies Inc., Santa Clara, CA, USA) with luminescence. All assays were performed in accordance with the manufacturers’ instructions.

Whole body insulin sensitivity was calculated at each visit using the Matsuda index (Matsuda & DeFronzo, 1999) as follows:

$$\mathrm{IS}I_{\mathrm{MATSUDA}}=10,000/\surd \left(G_{0}\times I_{0}\right)\times \left(G_{\mathrm{MEAN}}\times I_{\mathrm{MEAN}}\right)$$

where ISI is insulin sensitivity index; *G*_0_ is baseline (pre‐drink, i.e., min 0) glucose (mg/dL); *I*_0_ is baseline insulin (μIU/mL); *G*_MEAN_ is average glucose levels from 30−120‐min post‐drink‐consumption; and *I*_MEAN_ is average insulin levels from 30−120‐min post‐drink‐consumption. The insulinogenic index was calculated as an indicator of the efficiency of glucose disposal in the early phase of insulin secretion using the formula: $\left(I_{30}-I_{0}\right)/\left(G_{30}-G_{0}\right),$where *I*_0_ is baseline insulin (μIU/mL); *I*_30_ is insulin at 30 min post‐consumption; *G*_0_ is baseline glucose (mg/dL); and *G*_30_ is glucose at 30 min post‐consumption. The disposition index was assessed as the product of the ISI and insulinogenic index. The insulin area under the curve (AUC) and glucose AUC were also calculated using the trapezoidal rule (Jenkins et al., 2020). The liver insulin resistance index was calculated as the product of the insulin and glucose AUCs during the first 30 min of the oral glucose tolerance test (OGTT), while the muscle insulin sensitivity index was calculated as the dividend of the plasma glucose rate of decay from the peak to nadir value and the mean plasma insulin concentration during the OGTT (Abdul‐Ghani et al., 2007).

### 2.9 Blood pressure

Blood pressure was measured using an automated blood pressure cuff (OMRON 7 Series, BP‐7450, Omron Healthcare, Bannockburn, IL, USA) on the arm opposite to the one with the i.v. catheter. The average of two readings was taken at each time point and used for analyses.

### 2.10 Sample size estimation

We assessed the sample size necessary to determine the effect of SIT versus REB on the blood flow response (Δblood flow) by sex (i.e., condition × sex on Δblood flow), using a conservative Cohen's *f* of 0.35 based on a prior study (Hartman et al., 2021), a power of 0.8, an α of 0.05, two repeated measurements, and a correlation among repeated measures of 0.6. We determined that we would need 16 subjects in total (8 female/8 male) to answer this research question. To account for subject dropout, considering the time commitment associated with participation, we conservatively recruited 24 subjects. The majority of participants who enrolled were female and none dropped out; thus, all 24 subjects were included in the analyses.

### 2.11 Statistical analyses

Statistical analyses were performed using GraphPad Prism for macOS (v. 8.4.3, GraphPad Software, San Diego, CA, USA) and R (v. 4.4.0, R foundation for Statistical Computing, Vienna, Austria; GUI 1.80 Big Sur ARM build (8376)), and significance was set at *P* ≤ 0.05. Figures were made using GraphPad Prism for macOS (v. 8.4.3), R, and BioRender.com. All data were tested for normality and homogeneity using the Shapiro–Wilk test and Levene's test, respectively. Descriptive statistics are reported as means ± SD and all other data are presented as predicted (LS) mean ± standard error of difference, unless stated otherwise. Between‐condition (SIT vs. REB) differences in kcal consumed the day before experimental visits and between‐sex baseline characteristics were assessed using paired samples and Student's *t*‐test for independent samples, respectively. We examined the effects of REB versus SIT (a) in the intervention period alone (i.e., min −210−0) separately from the effects of REB versus SIT on (b) responses to the oral glucose challenge (i.e., increased circulating insulin, min 0−120) on all of our outcome variables. Sex was also included as an independent variable in the primary models for all analyses. There were missing data at random throughout the data set. Therefore, we utilized mixed effects models, which can handle missing data for analyses rather than using listwise deletion. For glucose, insulin, ET‐1, and perfusion data, data missingness was 2.1%, 4.2%, 3.5%, and 9%, respectively.

First, effects across the intervention period on gastrocnemius perfusion (−210 and 0 min), popliteal shear rate (−210, −120, −60 and 0 min), brachial artery blood flow (−210 and 0 min), glucose, insulin and ET‐1 concentrations (−210, −120, −60 and 0 min), and systolic (SBP) and diastolic (DBP) blood pressure (−210 and 0 min) were examined using three‐way (sex [female vs. male] × condition [REB vs. SIT] × time [all available time points from −210 to 0 min]) mixed‐effects models.

Next, three‐way (sex [female vs. male] × condition [REB vs. SIT] × time [all available time points from 0 to 120 min]) mixed effects models were used to examine the effects of the intervention on subsequent changes in gastrocnemius perfusion (0, 60, 90 and 120 min), brachial and popliteal artery blood flow (0, 30, 60, 90 and 120 min), glucose, insulin and ET‐1 concentrations (0, 30, 60, 90 and 120 min), and blood pressure (0, 60 and 120 min) in response to the oral glucose load. Two‐way mixed effects models (sex [female vs. male] × condition [REB vs. SIT]) were used to examine the effects of the intervention on ISI_MATSUDA_, the insulinogenic, disposition, hepatic insulin resistance and muscle insulin sensitivity indices, insulin area under the curve (AUC), and glucose AUC. Lower‐order mixed effects models and Tukey's or Šidák‐corrected post‐hoc tests were used to decompose interactions. Similarly, to examine main effects, mixed effects models and/or Tukey's or Šidák‐corrected comparisons were used to examine differences among the corresponding marginal means. Repeated measures correlation coefficients (*r*_rm_) were used to examine the association of insulin and glucose concentrations with gastrocnemius perfusion immediately before (0 min) and after (60, 90 and 120 min) consumption of the oral glucose load during the SIT and REB conditions using the rmcorr package (v. 0.6.0) in R. Associations between insulin and glucose AUCs and insulin sensitivity/resistance indices and changes in perfusion were examined using Pearson's correlation coefficients, in accordance with prior studies examining changes in perfusion in response to oral glucose loads (Parker et al., 2020).

## 3 RESULTS

### 3.1 Lifestyle controls

Within sex, participants consumed similar calories on the days before each experimental visit (*P* ≥ 0.361). Menstrual cycle phase was the same at both experimental visits for 12 of the 16 female participants. One participant reported having no cycle due to long‐acting contraceptive use. For those who attended visits at different phases of their cycle, all three participants completed the SIT condition in the luteal phase and the REB condition in the follicular phase. Of the 16 female participants included in this study, 11 did not take any hormone contraceptives, two took oral contraceptives and three had an intrauterine device (IUD).

### 3.2 Effects during the intervention period

#### 3.2.1 Popliteal shear rate

**FIGURE 2:**
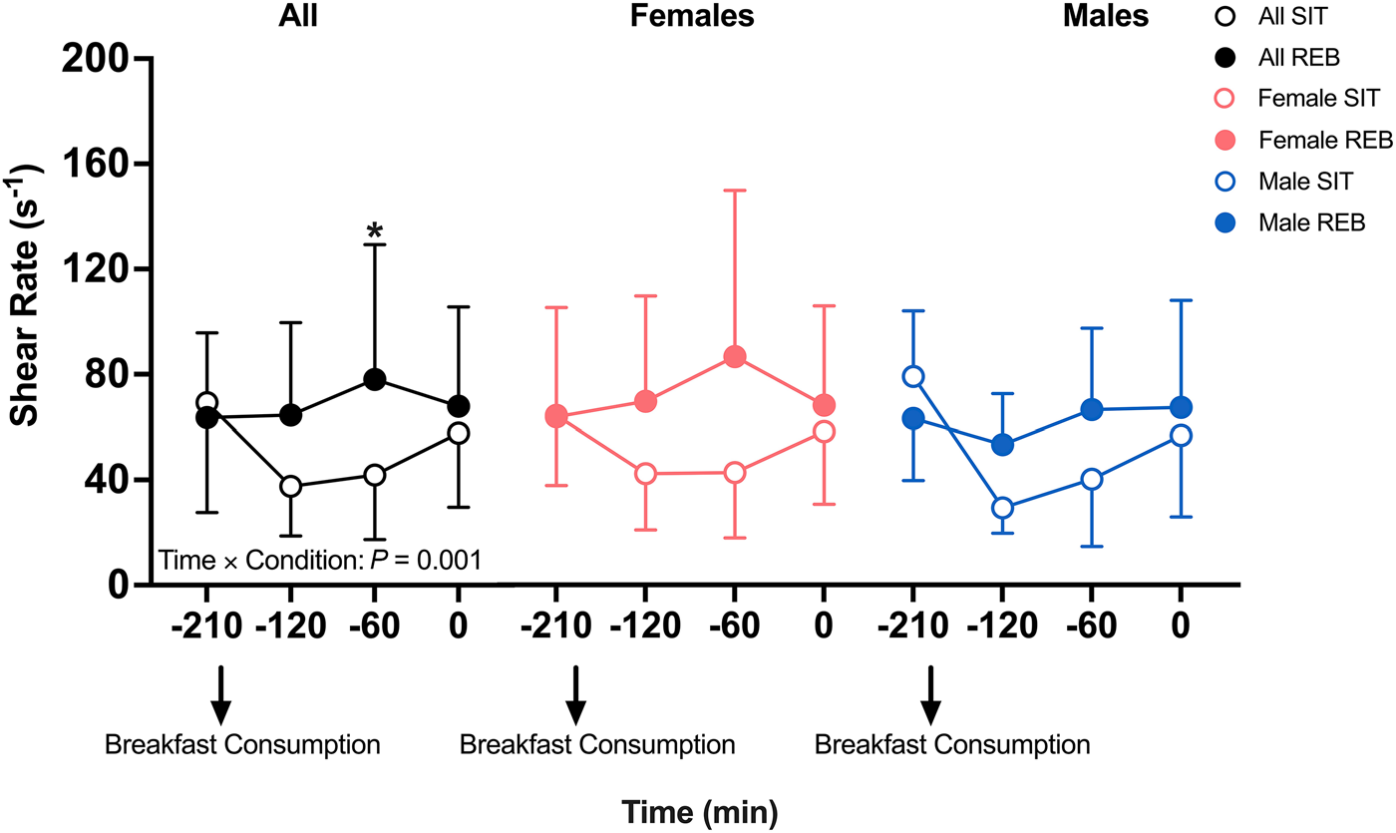
Popliteal shear rate during a 3‐h sedentary period with (REB) or without (SIT) resistance exercise breaks performed every 30 min. Data are from 24 (16 female and 8 male) participants and are displayed as means ± SD. Significance was set at *P* < 0.05. *Significantly different from the same time point in the SIT condition in all participants.

Popliteal shear rates during the intervention period are shown in Figure 2. A significant condition × time interaction was observed for popliteal shear rates during the intervention period (*P* = 0.0005). Follow‐up analyses revealed that REB significantly increased popliteal artery shear rate compared to SIT at −120 min (+23.6 ± 8.4 s^−1^, *P* = 0.025) and −60 min (+34.8 ± 9.0 s^−1^, *P* = 0.001), and this effect was independent of sex. No differences were observed between conditions at −210 min (*P *= 0.90) or 0 min (*P *= 0.47). No other significant interactions or main effects were observed.

#### 3.2.2 Gastrocnemius perfusion

**FIGURE 3:**
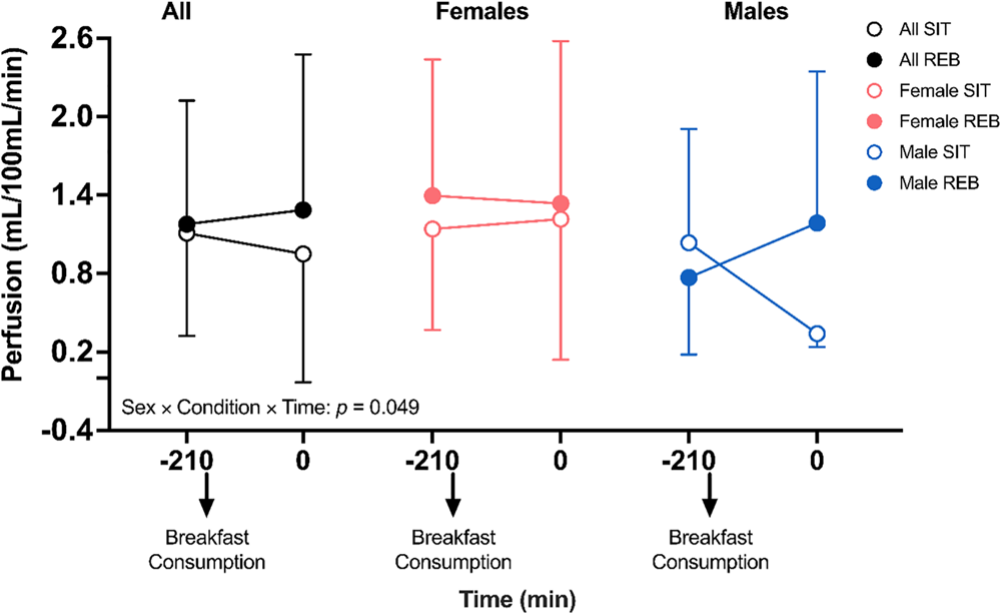
Gastrocnemius perfusion from before (−210 min) to after (0 min) a 3‐h sedentary period with (REB) or without (SIT) resistance exercise breaks performed every 30 min. Data are from 24 (16 female and 8 male) participants. A significant time × sex × condition interaction was observed. However, after performing follow‐up, sex‐stratified analyses no significant effects were observed (*P *≥ 0.11). Data are displayed as mean ± SD, were analysed using mixed‐effects analysis and post‐hoc tests as appropriate, and significance was set at *P* < 0.05.

Perfusion data from −210 to 0 min are displayed in Figure 3. A significant sex × condition × time interaction was observed for gastrocnemius perfusion across the intervention period (*P* = 0.049). However, in sex‐stratified follow‐up analyses, there were no significant condition × time effects in males (*P* = 0.114) or females (*P* = 0.617).

#### 3.2.3 Vascular and metabolic outcomes

Vascular and metabolic data collected during the intervention period are shown in Table 2. There was a significant main effect of condition for glucose, where glucose was greater in REB than SIT across the intervention period (+5.0 ± 2.0 mg/dL; *P *= 0.024). There was a main effect of time for insulin, which increased from −210 to −120 min (+36 ± 6 μIU/mL, *P* < 0.001), and then decreased from −120 to −60 min (−21 ± 6 μIU/mL, *P* = 0.005) and 0 min (−33 ± 6 μIU/mL, *P* < 0.001) to concentrations that were similar to −210 min (*P *≥ 0.08). There was also a significant condition × sex interaction for insulin, but no significant differences were observed between conditions for either sex in follow‐up analyses (*P* ≥ 0.15). There was a significant main effect of sex for brachial blood flow, where males had significantly greater blood flow than females irrespective of condition or time (+62.19 ± 22.35 mL/min; *P *= 0.009). No significant interactions (*P* ≥ 0.25) or main effects (*P* ≥ 0.140) were observed for ET‐1. Lastly, there was a significant time main effect for DBP, where DBP decreased from −210 to 0 min regardless of sex or condition (−2.14 ± 0.84 mmHg; *P *= 0.018). No other significant interactions or main effects were observed.

**TABLE 2:** Vascular and metabolic outcomes from pre‐ (–210 min) to post‐intervention period (0 min; e.g., baseline).

Variable	Females				Males				*P*						
	–210		0		–210		0								
	REB	SIT	REB	SIT	REB	SIT	REB	SIT	Time × Sex × Condition	Sex × Condition	Time × Condition	Time × Sex	Time	Sex	Condition
Glucose (mg/dL)	93.7 ± 3.2	93.0 ± 3.1	88.6 ± 2.6	85.1 ± 4.3	97.1 ± 2.5	91.4 ± 3.7	85.5 ± 1.9	82.0 ± 2.5	0.727	0.777	0.470	0.864	0.09	0.734	**0.024**
Insulin (μIU/mL)	15.5 ± 3.0	13.6 ± 2.7	18.0 ± 4.9	25.5 ± 7.7	19.2 ± 4.6	20.7 ± 5.3	20.9 ± 6.2	15.3 ± 3.8	0.198	**0.009**	0.723	0.636	**<0.001**	0.701	0.853
ET‐1 (pg/mL)	3.06 ± 0.38	3.04 ± 0.38	2.99 ± 0.34	2.97 ± 0.43	2.15 ± 0.49	2.16 ± 0.45	2.00 ± 0.45	2.13 ± 0.44	0.949	0.250	0.869	0.971	0.660	0.140	0.684
Brachial BF (mL/min)	78.0 ± 17.6	69.8 ± 17.6	74.2 ± 18.7	64.2 ± 18.7	126.6 ± 36.7	133.6 ± 36.7	81.7 ± 25.8	122.8 ± 25.8	0.492	0.208	0.535	0.401	0.238	**0.002**	0.566
SBP (mmHg)	107.6 ± 1.4	108.5 ± 1.4	107.4 ± 1.4	106.9 ± 1.4	111.4 ± 2.0	110.8 ± 2.0	111.5 ± 2.1	109.5 ± 2.1	0.973	0.508	0.267	0.843	0.420	0.391	0.602
DBP (mmHg)	69.3 ± 1.4	71.9 ± 1.4	67.9 ± 1.4	68.3 ± 1.4	66.1 ± 1.9	66.6 ± 1.9	65.4 ± 2.0	64.7 ± 2.0	0.657	0.484	0.146	0.534	**0.048**	0.291	0.531

*Note*: Data are presented here as predicted (LS) mean ± SE. *P*‐values are from three‐way linear mixed models and significant *P*‐values (<0.05) are shown in bold. Abbreviations: BF, blood flow; DBP, diastolic blood pressure; ET‐1, endothelin‐1; SBP, systolic blood pressure.

### 3.3 Responses to oral glucose loading

#### 3.3.1 Glucose and insulin

**FIGURE 4:**
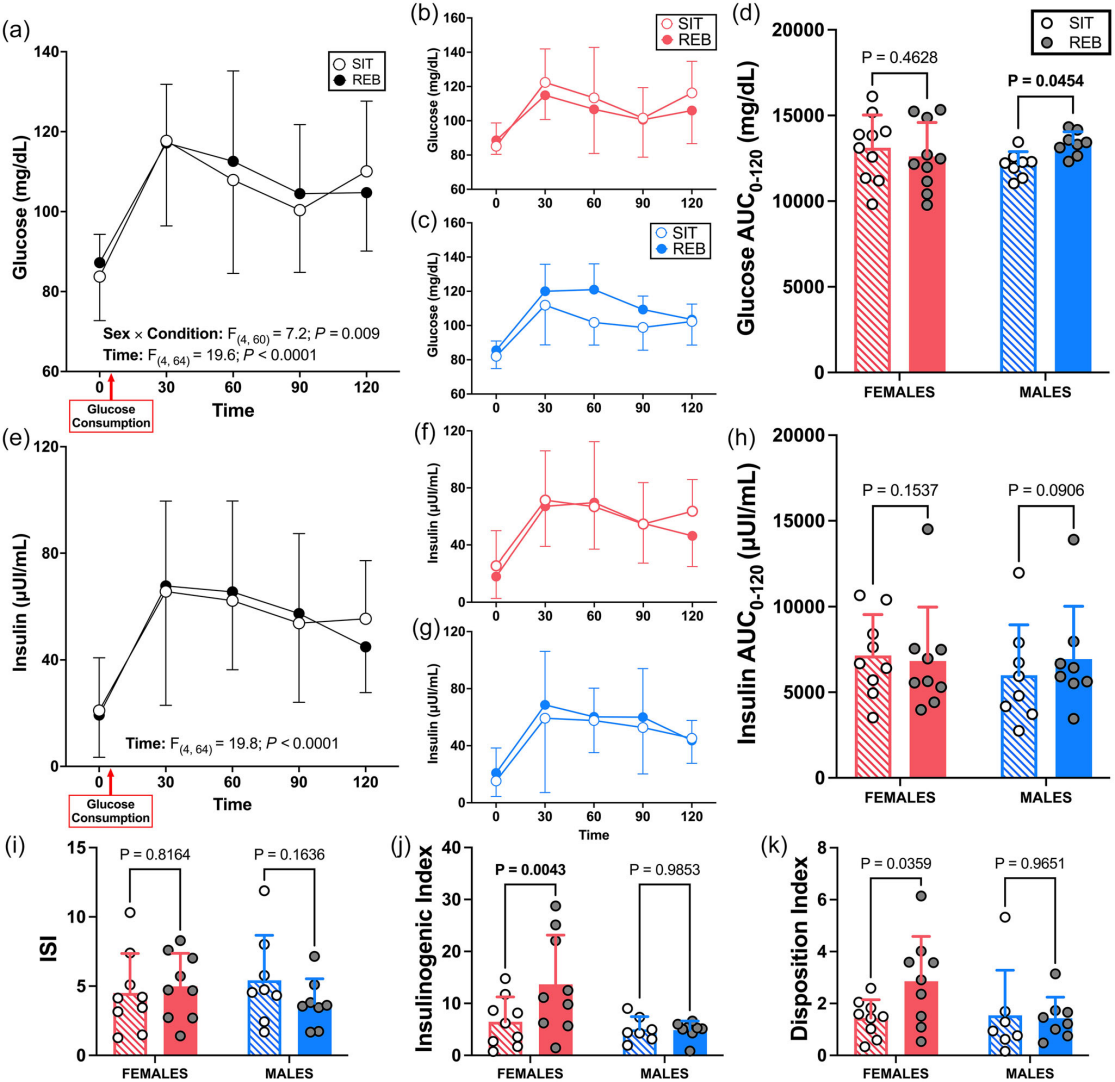
Glucose and insulin responses to a 75 g oral glucose load (min 0−120) following prolonged sitting (SIT) or prolonged sitting with resistance exercise breaks performed every 30 min (REB) in 18 (10 female and 8 male) participants. Glucose and insulin responses to the OGTT are shown for all subjects combined (a, e), and for female (b, f) and male (c, g) participants separately. Glucose and insulin AUCs (d, h), Matsuda insulin sensitivity index (ISI; i), insulinogenic index (j), and disposition index (k) are also presented in female (*n* = 9) and male (*n* = 8) participants. Data are displayed as means ± SD and significance was set at *P* < 0.05.

Glucose and insulin responses to the oral glucose load are presented in Figure 4. There were no significant interaction effects (all *P* ≥ 0.13) or main effects of condition (*P *= 0.88) or sex (*P* = 0.44) in response to the oral glucose load for insulin. Independent of condition and sex, insulin concentrations increased from 0 to 30 min (+48 ± 6 μIU/mL, *P* < 0.0001) and remained elevated through the rest of the postprandial period (+31 ± 6 to 47 ± 6 μIU/mL, all *P *< 0.0001). There was a significant condition × sex interaction (*P* = 0.009) and a main effect of time (*P* < 0.0001) for glucose concentrations in response to the oral glucose load. In post‐hoc analyses, there were no differences in average glucose concentrations for REB versus SIT in males (99 ± 6 vs. 108 ± 5 mg/dL, *P* = 0.06) or females (104 ± 15 vs. 108 ± 15 mg/dL, *P* = 0.38). Independent of condition and sex, glucose concentrations increased from 0 to 30 min (+32 ± 4 mg/dL, *P *< 0.0001) and then decreased from 60 to 90 min (−8 ± 4 mg/dL, *P* = 0.046), but remained elevated above baseline levels across the entire post‐prandial period (all *P *< 0.0001).

There was a condition × sex interaction for both glucose (*P* = 0.018) and insulin (*P* = 0.012) AUC_0–120_. The glucose AUC_0–120_ was greater in REB than in SIT (±1217 ± 484 mg/dL, *P* = 0.045) for males, but was not different between REB and SIT (−498 ± 433 mg/dL, *P* = 0.46) for females. In post‐hoc analyses, there were no differences in insulin AUC_0–120_ for REB versus SIT in males (+939 ± 429 μIU/mL, *P* = 0.09) or females (−807 ± 428 μIU/mL, *P* = 0.15). No significant condition × sex interaction (*P *= 0.11), main effect for sex (*P *= 0.97), or main effect for condition (*P *= 0.37) was observed for the Matsuda Index. There was a significant condition × sex interaction for the insulinogenic index (*P *= 0.018), which was greater in REB than SIT in females (+7.3 ± 1.9, *P* = 0.004), but not in males (−0.3 ± 2.1, *P* = 0.99). No significant effects were observed for the hepatic insulin resistance (all *P* ≥ 0.26) or muscle insulin sensitivity (all *P* ≥ 0.06) indices.

#### 3.3.2 Endothelin‐1

**FIGURE 5:**
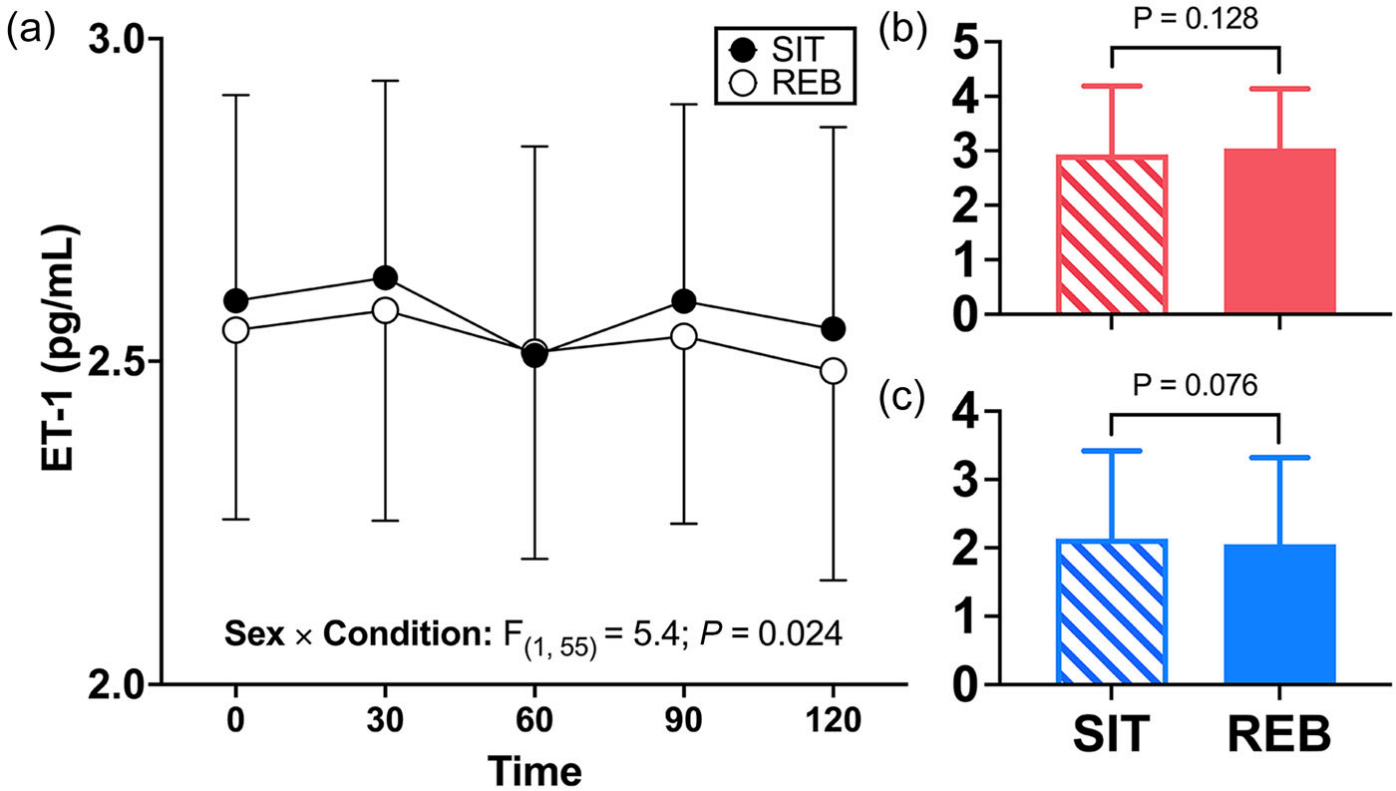
(a) Endothelin‐1 (ET‐1) responses to a 75 g oral glucose load (min 0−120) following prolonged sitting (SIT) or prolonged sitting with resistance exercise breaks performed every 30 min (REB) in 18 participants. A significant sex × condition interaction was observed (*P *= 0.024). (b, c) Follow‐up condition effects are shown for females (b; *n* = 10) and males (c; *n* = 8), which were not significant for either sex. Data are displayed as means ± SD, were analysed using mixed‐effects analysis (missing values = 9 of 180) and post‐hoc tests as appropriate, and significance was set at *P* < 0.05.

ET‐1 data are displayed in Figure 5. A significant sex × condition interaction was observed for ET‐1 (*P *= 0.024). However, in follow‐up analysis, the effect of the condition was not significant in females (*P* = 0.128; Figure 5b) or males (*P *= 0.076; Figure 5c). No other interactions (*P *> 0.42) or main effects (*P *> 0.11) were observed.

#### 3.3.3 Gastrocnemius perfusion

**FIGURE 6:**
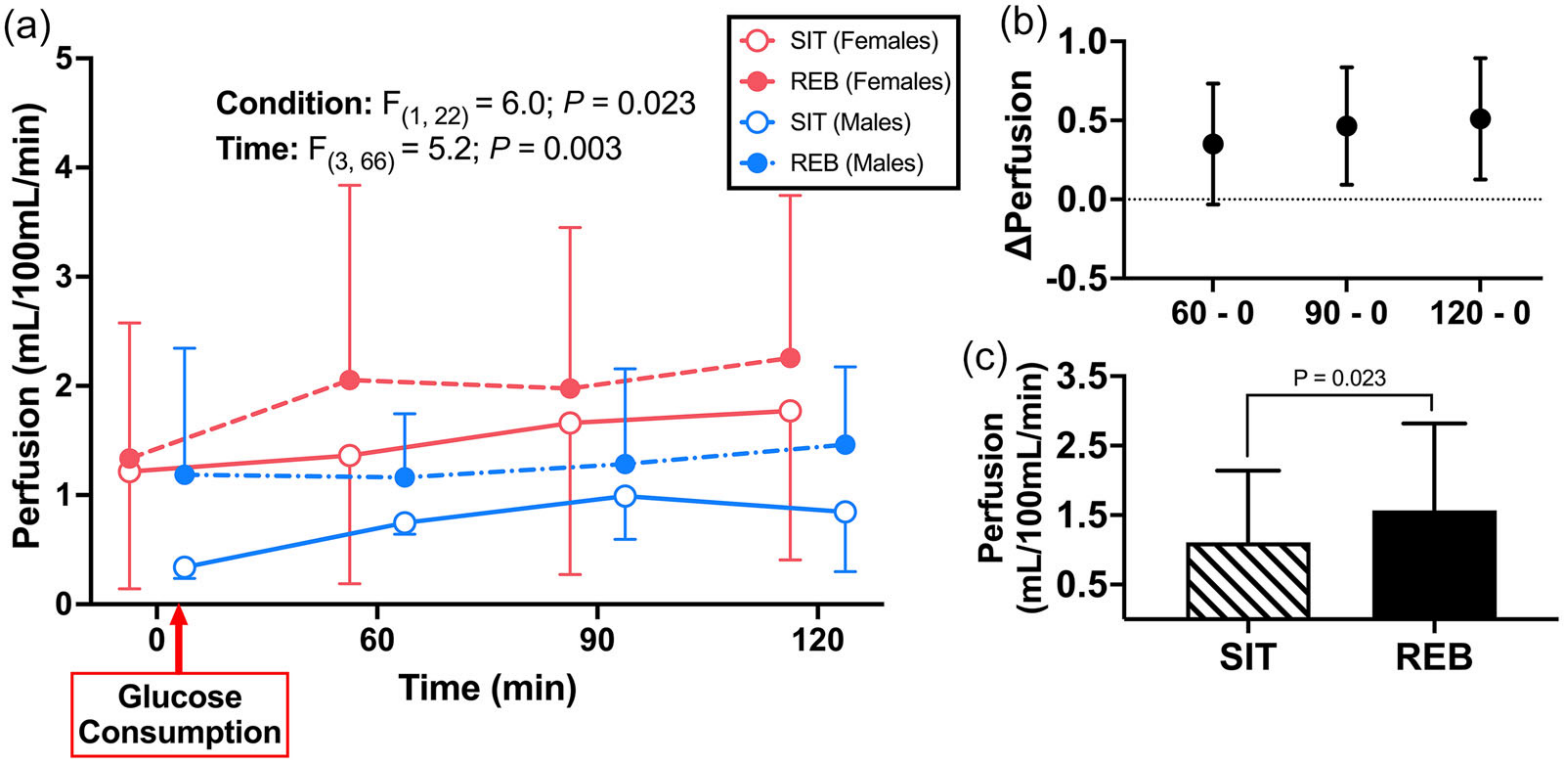
(a) Gastrocnemius perfusion responses to oral glucose loading (min 0−120) in female (*n* = 16) and male (*n* = 8) participants following prolonged sitting (SIT) or prolonged sitting with resistance exercise breaks (REB) performed every 30 min. (b, c) Significant main effects of time (*P* = 0.003; b) and condition (*P* = 0.023; c) were observed. Perfusion increased from 0 to 90 (*P* = 0.009) and 0 to 120 min (*P* = 0.005) and was greater in REB compared to SIT (*P* = 0.023). Data are displayed as means ± SD, were analysed using mixed‐effects analysis (missing values = 18 of 192), and significance was set at *P* < 0.05.

Gastrocnemius perfusion data from 0−120 min are displayed in Figure 6. There were no significant interactions (*P* ≥ 0.29), nor was there a main effect of sex (*P *= 0.09) for gastrocnemius perfusion. However, there were main effects of both time (*P *= 0.003) and condition (*P *= 0.023). Perfusion was significantly greater after REB compared to SIT (1.57 vs. 1.11 mL/100 mL/min, *P *= 0.023), and significantly increased from 0 to 90 min (+0.46 ± 0.14 mL/100 mL/min, *P* = 0.009), and 120 min (+0.51 ± 0.15 mL/100 mL/min, *P *= 0.005).

#### 3.3.4 Popliteal artery blood flow

**FIGURE 7:**
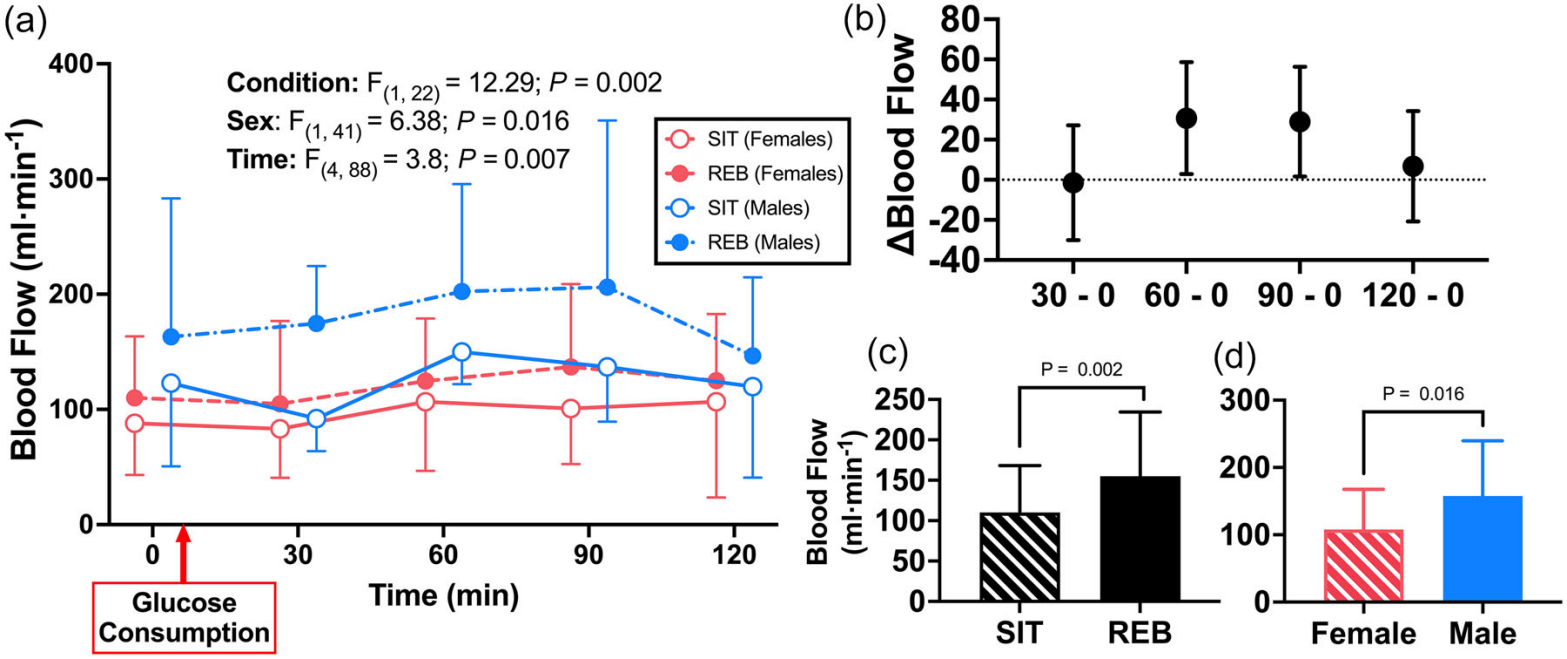
(a) Popliteal artery blood flow responses to oral glucose loading (min 0−120) in female (*n* = 16) and male (*n* = 8) participants following prolonged sitting (SIT) or prolonged sitting with resistance exercise breaks (REB) performed every 30 min. (b–d) Significant main effects of time (*P* = 0.007; b), condition (*P* = 0.002; c), and sex (*P* = 0.016; d) were observed. Blood flow increased from 0 to 60 and 90 min (*P* = 0.026 and 0.034, respectively) and was greater in REB compared to SIT. Popliteal artery blood flow was also greater in males compared to females. Data are displayed as mean ± SD, were analysed using mixed‐effects analysis (missing values = 47 of 240), and significance was set at *P* < 0.05.

Popliteal artery blood flow data from 0 to 120 min are presented in Figure 7. No significant interactions were observed for popliteal blood flow (*P* ≥ 0.16). However, significant main effects of time (*P* = 0.007), condition (*P* = 0.002) and sex (*P* = 0.016) were observed. Popliteal blood flow increased from 0 to 60 min (+30.8 ± 11 mL/min, *P* = 0.026) and 90 min (+28.95 ± 11 mL/min, *P* = 0.034), and was greater at REB versus SIT (155.3 vs. 110.2 mL/min, *P* = 0.001). Popliteal blood flow was also greater in males compared to females (157.6 vs. 107.8 mL/min, *P* = 0.016).

#### 3.3.5 Brachial artery blood flow

A significant sex × condition interaction was observed for brachial blood flow (*P *= 0.026). However, in follow‐up analyses there was no significant condition effect in either males (*P* = 0.20) or females (*P* = 0.098). Brachial artery blood flow was significantly greater in males than females in REB (*P* = 0.003). No other significant interactions or main effects were observed.

#### 3.3.6 Blood pressure

A significant sex × time interaction was observed for SBP (*P *= 0.010) where males’ SBP increased during the 0−120‐min time‐period (*P* = 0.032) and females’ remained unchanged (*P* = 0.114). Post‐hoc analyses revealed that males’ SBP was higher at 120 compared to 0 min (+5.13 ± 1.75 mmHg, *P* = 0.028). No other significant interactions or main effects were observed for SBP (*P* ≥ 0.208). There were no significant interactions or main effects observed when examining DBP (*P* ≥ 0.069).

#### 3.3.7 Correlations

**FIGURE 8:**
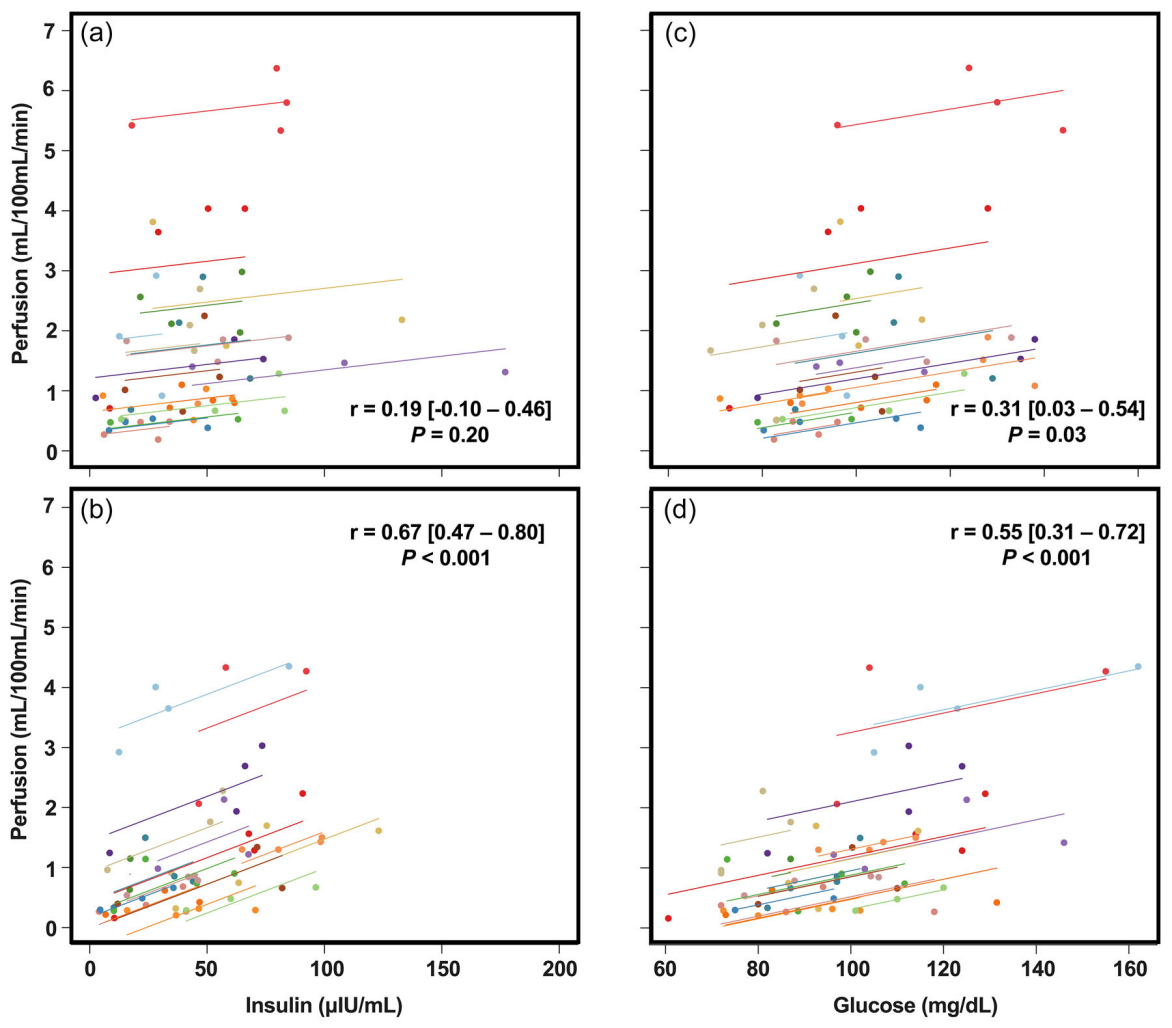
Within‐subject, repeated measures correlations for gastrocnemius perfusion versus insulin in the REB (a) and SIT (b) conditions, and for gastrocnemius perfusion versus glucose REB (c) and SIT (d) conditions. Paired perfusion and metabolic data are from 0, 60, 90 and 120 min measurement points.

The repeated measures correlations (Figure 8) indicated that within‐individual increases in insulin were associated with increases in perfusion in SIT (*r*_rm_ = 0.67, *P* < 0.001) but not REB (*r*_rm_ = 0.19, *P* = 0.20), whereas within‐individual increases in glucose were associated with increases in perfusion in both SIT (*r*_rm_ = 0.55, *P* < 0.001) and REB (*r*_rm_ = 0.31, *P* = 0.03). Between‐subject correlation analyses indicated that, in SIT, insulin AUC was not related to changes in perfusion observed at 60 min (*r* = 0.05, *P *= 0.65) or 90 min (*r* = 0.35, *P *= 0.20), and was moderately but not significantly related to the changes in perfusion observed at 120 min (*r* = 0.50, *P *= 0.07). Glucose AUC was strongly and significantly related to the changes in perfusion observed at 60 min (*r* = 0.58, *P *= 0.03), but not 90 min (*r* = 0.15, *P *= 0.59) or 120 min (*r* = 0.34, *P *= 0.22). There were no relations between the liver resistance index and perfusion at any time point (*r* ≤ 0.32, *P* > 0.25), while there was a moderate but non‐significant relation between the muscle insulin sensitivity index and perfusion at 120 min (*r* = −0.49, *P* = 0.06). In REB, insulin AUC was moderately but not significantly related to the changes in perfusion observed at 60 min (*r* = −0.47, *P *= 0.08), 90 min (*r* = −0.39, *P *= 0.12), and 120 min (*r* = −0.35, *P *= 0.21). Glucose AUC was weakly and not significantly related to the changes in perfusion observed at any time point (*r* values −0.06 to −0.23, *P* values 0.39−0.84). However, the liver resistance index was significantly and inversely related to changes in perfusion at 60 min (*r* = −0.58, *P* = 0.017) and 90 min (*r* = −0.51, *P* = 0.029), but not 120 min (*r* = −0.42, *P* = 0.10).

## 4 DISCUSSION

The most salient finding from the current study is that 3‐min REB, performed every 30 min throughout an otherwise sedentary 3‐h period, was capable of augmenting skeletal muscle blood flow in response to an oral glucose load following prolonged sitting. Notably, this vasodilatory effect appeared to be local to the that were muscles active during REB, as REB improved gastrocnemius perfusion and popliteal artery blood flow, but not brachial artery blood flow postprandially. We also observed significant, moderate repeated measures associations of both insulin and glucose with perfusion in response to the oral glucose load in the SIT condition, whereas there was only a significant, small within‐subject association of glucose with perfusion in the REB condition. Finally, while the vasodilatory effects were largely independent of sex in the current study, there was evidence that sex influenced the acute effects of prolonged sitting on perfusion and the effect of REB on postprandial glucose metabolism and circulating ET‐1 concentrations.

As sitting‐induced impairments in postprandial vascular function have been identified as a likely link between sedentary behaviour and increased CVD and all‐cause mortality incidence (Walsh et al. 2019), the current study presents a potentially effective strategy to offset these risks. This study adds to the growing body of literature investigating potentially feasible strategies to improve cardiometabolic function in the face of prolonged sedentary time. For example, Climie et al. observed that the shear rate was increased and FMD% was preserved by interrupting sitting every 30 min using 3‐min REB during prolonged (5‐h) sitting in adults who were overweight or obese (Climie et al., 2018). Walsh et al. demonstrated that augmenting shear stress using passive leg heating during prolonged sitting promotes an increase in insulin‐stimulated blood flow (Walsh et al., 2019). Our findings are in agreement with, and build upon, the findings of both Climie et al. (2018) and Walsh et al. (2019). First, we showed that REB successfully elicited increases in popliteal artery shear rate during the otherwise sedentary period (Figure 2). Subsequently, while insulin increased to the same degree in REB and SIT following glucose ingestion, REB augmented increases in gastrocnemius muscle perfusion and popliteal artery blood flow by 42% (*P *= 0.021) and 41% (*P* = 0.002) relative to SIT, respectively (Figures 6 and 7). Given that blunted vasodilatation in response to vasodilatory hormones (e.g., insulin) and/or postprandial vasodilatation occur early in the development, or even precede the onset, of CMD (Eringa et al., 2007; Katakam et al., 1998; Olver et al., 2019a), impaired postprandial vasomotor function has been proposed as a mechanism linking sedentary behaviour with CMD. Therefore, our data indicate that periodic REBs performed during a prolonged sedentary period are capable of augmenting skeletal muscle blood flow following oral glucose ingestion and thus may be an effective strategy to reduce CMD risk associated with prolonged sedentary behaviour.

Our data indicate that both insulin and glucose concentrations were positively associated with perfusion within individuals in response to the OGTT following the SIT condition (Figure 8b,d). Examination of the inter‐individual associations between insulin and glucose AUCs with the changes in perfusion indicated that, in SIT, there was a significant, strong positive relation between glucose AUC and the change in perfusion at 60 min in response to the OGTT. Prior evidence has indicated that acute hyperglycaemia induced by oral glucose loading impairs skeletal muscle perfusion even while causing marked increases in insulin concentrations, conduit artery vasodilatation and blood flow, and decreased peripheral resistance (Parker et al., 2020; Russell et al., 2018). These studies have also reported inverse associations between postprandial insulin and glucose and microvascular blood flow (Parker et al., 2020; Russell et al., 2018). Indeed, Russell et al. (2018) reported significant inverse associations between changes in blood glucose concentrations and microvascular blood flow in response to oral glucose and liquid mixed meal ingestion. However, closer examination indicated that hyperinsulinaemia was related to greater microvascular blood flow when concomitant increases in glucose concentrations were moderate and less than 2.4 mM (∼43 mg/dL) above fasting (Russell et al., 2018). Thus, our findings of a significant positive intra‐individual relation between insulin and glucose with perfusion in SIT may be explained by the fact that, on average, the peak postprandial glucose excursion was 43 mg/dL following SIT, and thus the degree of hyperglycaemia may not have been sufficient to impair skeletal muscle microvascular responsiveness as observed in these prior studies (Parker et al., 2020; Russell et al., 2018).

In the REB condition, only glucose concentrations were significantly, but weakly, associated with perfusion within individuals (Figure 8c), while there was an inverse moderate, but non‐significant, relation between insulin AUC and changes in perfusion at 60 min (*r* = −0.47). These findings are in contrast to our observations in the SIT condition and were unexpected, as we anticipated that insulin concentrations would be significantly associated with vasodilatory responses following glucose consumption and that this relation would be positive and strongest in REB, reflective of improved vasodilatory responsiveness to insulin. It is notable and unexpected that changes in perfusion at both 60 and 90 min were strongly and inversely related to the hepatic insulin resistance index in REB. This finding may suggest the presence of a mediator that improves hepatic insulin sensitivity and enhances skeletal muscle perfusion in response to oral glucose loading, but this is highly speculative and to our knowledge has not previously been described. However, recent work provides compelling evidence that the gut‐derived hormones gastric inhibitory polypeptide (GIP) and glucagon‐like peptide‐1 (GLP‐1) play a critical role in determining the microvascular response to oral glucose ingestion, perhaps more so than hyperglycaemia per se (Roberts‐Thomson et al., 2022). Systemic GLP‐1 infusion at postprandial levels has been shown to increase skeletal muscle blood volume and flow by ∼30%, and this effect is not augmented by superimposition of insulin infusion (Wang et al., 2020). GLP‐1 has also been described as a regulator of hepatic glucose production that increases hepatic insulin sensitivity (Yang et al., 2017). It has also recently been reported that intermittent walking during prolonged sedentary periods augments the postprandial GLP‐1 response without significantly influencing the GIP response in individuals with central overweight/obesity (Chen et al., 2022). Thus, it is plausible that both the inverse relation observed between hepatic insulin resistance and changes in perfusion and the lack of association between insulin and perfusion observed in REB may be explained by augmented secretion of an unobserved hormone with vasoactive and hepatic insulin‐sensitizing actions, such as GLP‐1. Unfortunately, we are limited in our ability to draw any concrete conclusions because we did not measure the incretins in this study. It is also important to note that none of the prior studies described have examined intra‐individual associations to understand whether changes in insulin or glucose are related to changes in perfusion within individuals across the postprandial period, but have rather deployed interindividual correlation analysis that may also partially explain differences observed herein versus in these prior studies.

One novel aspect of our study was determining whether the ability of REB to improve leg blood flow responses to an oral glucose load following prolonged sitting is dependent on sex. Emerging evidence suggests that the female sex may confer protection against various lifestyle‐induced impairments in vascular endothelial function (Manrique‐Acevedo et al., 2023; Morishima et al., 2020; Vranish et al., 2017). For example, Smith et al. (2022) recently observed decreased insulin‐stimulated blood flow in males, but not females, after completing a 10‐day obesogenic lifestyle intervention which included consumption of six cans of sugar‐sweetened beverages per day and a reduction in the movement to <5000 steps/day. Vranish et al. (2017) reported that prolonged (3‐h) sitting‐induced reductions in popliteal artery FMD% in males but not females, whereas microvascular reactivity was reduced similarly in both males and females (Vranish et al., 2017). While the effect of condition on blood flow response was not dependent on sex in the present study, there was a significant sex × condition interaction for ET‐1 concentrations in response to glucose ingestion. Follow‐up analysis indicated that there was no effect of the condition among females, but there was a tendency for ET‐1 concentrations to be greater following SIT than REB among males (*P *= 0.076). Notably, we also observed a sex × condition interaction for microvascular perfusion before and after the 3 h of prolonged sitting (SIT) compared to prolonged sitting with REB. Qualitatively, perfusion appeared to be more markedly reduced by SIT in males than females (Figure 3), perhaps suggesting that males may be more susceptible to reductions in muscle perfusion during prolonged sedentary periods. However, similar to ET‐1, no significant differences were observed in post‐hoc analyses, probably as a result of inadequate statistical power in follow‐up analyses. Finally, REB promoted an increase in glucose AUC in males only (Figure 4d), and the insulinogenic index was improved in REB in females only (Figure 4j). Together, these data indicate that there may be sex‐dependent effects of prolonged sitting and/or exercise breaks that should be explored in future studies with larger samples balanced for sex.

A few studies have been performed to gain a better understanding of the mechanism(s) by which prolonged sitting elicits impairments in postprandial vasodilatory function. In vitro and *ex vivo* evidence supports the hypothesis that decreased shear stress causes aberrant insulin signalling at the level of the vascular endothelium (Walsh et al., 2019), indicating that mechanotransduction of laminar shear stress is necessary to keep the vasodilatory signalling of insulin intact. Importantly, skeletal muscle blood flow is intricately connected to the metabolic demand of the tissue, which is dependent on the level of skeletal muscle activation during exercise, and adaptations occur primarily in arteries supplying tissues that undergo significant increases in activity (Olver et al., 2019b, 2016; Sun et al., 1998). Therefore, we deployed the use of lower body exercises with measurement of lower (i.e., gastrocnemius perfusion and popliteal artery blood flow) and upper body (i.e., brachial artery blood flow) blood flow to test the hypothesis that improvements in the blood flow response to oral glucose ingestion in response to REB would be localized to the vessels supplying muscles which were activated during REB. In accordance with our hypothesis, we observed improvements in the blood flow response to oral glucose ingestion in the lower, but not upper body. Therefore, our findings suggest that REB‐mediated improvements in blood flow following glucose ingestion are localized to the arteries supplying skeletal muscle tissues that were recruited by the exercises. These findings suggest that exercises which activate other major muscle groups could potentially also be deployed to promote widespread, systemic improvements, but this has not been tested. Furthermore, it is plausible that increasing the intensity of exercise breaks may promote more dramatic, widespread effects, but future studies will be necessary to test this hypothesis. Finally, as we did not measure blood flow response to glucose ingestion at baseline (i.e., before beginning the SIT or REB condition), we do not know if blood flow changed in either artery in question within conditions. Therefore, it is possible that we observed no improvement in brachial artery blood flow responses after REB due to a lack of impairment in this artery in response to SIT, as prior research shows impairments in popliteal, but not brachial, artery function (FMD%) in response to prolonged sitting (Restaino et al., 2015). Future studies will be needed to explore this hypothesis in the context of meal‐induced changes in regional blood flow.

There are several important limitations of the current study. First, efforts were made to examine female participants at the same stage within their menstrual cycle to avoid any possible effects of cycle‐related hormone fluctuations on vascular function (Wenner & Stachenfeld, 2020), but we did not directly measure oestrogen and progesterone levels in the blood, and thus cannot confirm if we accomplished this goal. Second, we chose to provide all subjects with the same standardized breakfast. Therefore, on average, female participants consumed more calories relative to their bodyweight than males. Future studies should consider providing breakfasts that are standardized to bodyweight to avoid potential confounding by creating differences in energy balance between subjects. In addition, no prior studies were available to estimate the effect of sex when planning this study. Therefore, we planned for a moderate sex‐related effect, but this was ultimately exploratory. The data included herein provides important preliminary evidence that can be used to guide sample size estimation for future studies examining the effect of sex. Finally, while we hypothesize that the shear stress stimulus was the mechanism by which REB ultimately promoted improvements in vasodilatation during the subsequent postprandial period, it cannot be ruled out that metabolic effects associated with skeletal muscle contraction and/or energy metabolism could have mediated these effects.

In conclusion, the current study found performing REB to be an effective strategy to augment the vasodilatory response to an oral glucose load following prolonged sitting in young sedentary males and females. As impaired postprandial vasodilatory function has been identified as a mechanistic explanation for the association between sedentary behaviour and the development of CMD, the findings herein present an effective strategy to maintain vascular health in populations who work primarily sedentary jobs. It will be critical for future studies to determine the feasibility and acceptability of this intervention in sedentary workers to understand whether it can be translated into practice. The current study should be replicated in older and clinical populations as the results observed herein may not be generalizable to populations other than young adults.

## AUTHOR CONTRIBUTIONS

The study was conceptualized and planned by Emily M. Rogers and Nathaniel D. M. Jenkins. Bethany Barone Gibbs, Lucas J. Carr, and Anna E. Stanhewicz critically evaluated and offered suggestions to improve study design. These changes were accepted and incorporated by Emily M. Rogers and Nathaniel D. M. Jenkins. Data collection and entry were completed by Emily M. Rogers, Nile F. Banks, Emma R. Trachta, and Morgan S. Wolf. Data analysis was performed by Emily M. Rogers, Nile F. Banks, Alexander C. Berry, and Nathaniel D. M. Jenkins. Emily M. Rogers was the primary creator of all figures and tables. Emily M. Rogers and Nathaniel D. M. Jenkins completed the original draft and all authors contributed to review and editing. Emily M. Rogers was responsible for project administration and Nathaniel D. M. Jenkins provided resources to complete the project. All authors approved the final version of the manuscript and agree to be accountable for all aspects of the work in ensuring that questions related to the accuracy or integrity of any part of the work are appropriately investigated and resolved. All persons designated as authors qualify for authorship, and all those who qualify for authorship are listed.

## CONFLICT OF INTEREST

Within the last 2 years, Emily M. Rogers and Nile F. Banks have received graduate assistant stipend funding from Woodbolt, LLC. Emily M. Rogers has received grant funding from the American College of Sports Medicine. Nile F. Banks and Nathaniel D. M. Jenkins have received grant funding from the National Strength and Conditioning Association. Nathaniel D. M. Jenkins has received grant funding from the National Institutes of Health (1R01HL167788), American Heart Association (24TPA1290435 and 18AIREA33960528), the Center for Integrative Research on Childhood Adversity (Award P20GM109097 through the NIGMS), the Injury Prevention Research Center (Award R49 CE003095 through the NCIPC/CDC), the National Institutes of Aging through the Research Network on Animal Models to Understand Social Dimensions of Aging, Woodbolt Distribution, LLC, and Applied Food Sciences, Inc, and has been the recipient of an NIH Clinical Research Loan Repayment Award. The present study does not constitute endorsement by ACSM. The results of the study are presented clearly, honestly, and without fabrication, falsification, or inappropriate data manipulation.

## Data Availability

The data that support the findings of this study are available from the corresponding author upon reasonable request.
